# Supplementary material for: Spatially resolved proteomic map shows that extracellular matrix regulates epidermal growth
Source: Nat Commun. 2022 Jul 11;13:4012. doi: 10.1038/s41467-022-31659-9 (PMC9273758; doi:10.1038/s41467-022-31659-9)
Supplement: Supplementary file 1 — Supplementary Information [file 41467_2022_31659_MOESM1_ESM.pdf]

Supplementary Information

Spatially resolved proteomic map shows that extracellular matrix regulates epidermal growth

Jun Li†*, Jie Ma†, Qiyu Zhang†, Huizi Gong, Dunqin Gao, Yujie Wang, Biyou Li, Xiao Li, Heyi Zheng, Zhihong Wu, Yunping Zhu*, Ling Leng†*

† These authors contributed equally.

* Correspondence to: lengling@pumch.cn (L.L.); zhuyunping@gmail.com (Y.Z.); lijun35@hotmail.com (J.L.).

Supplementary Figures

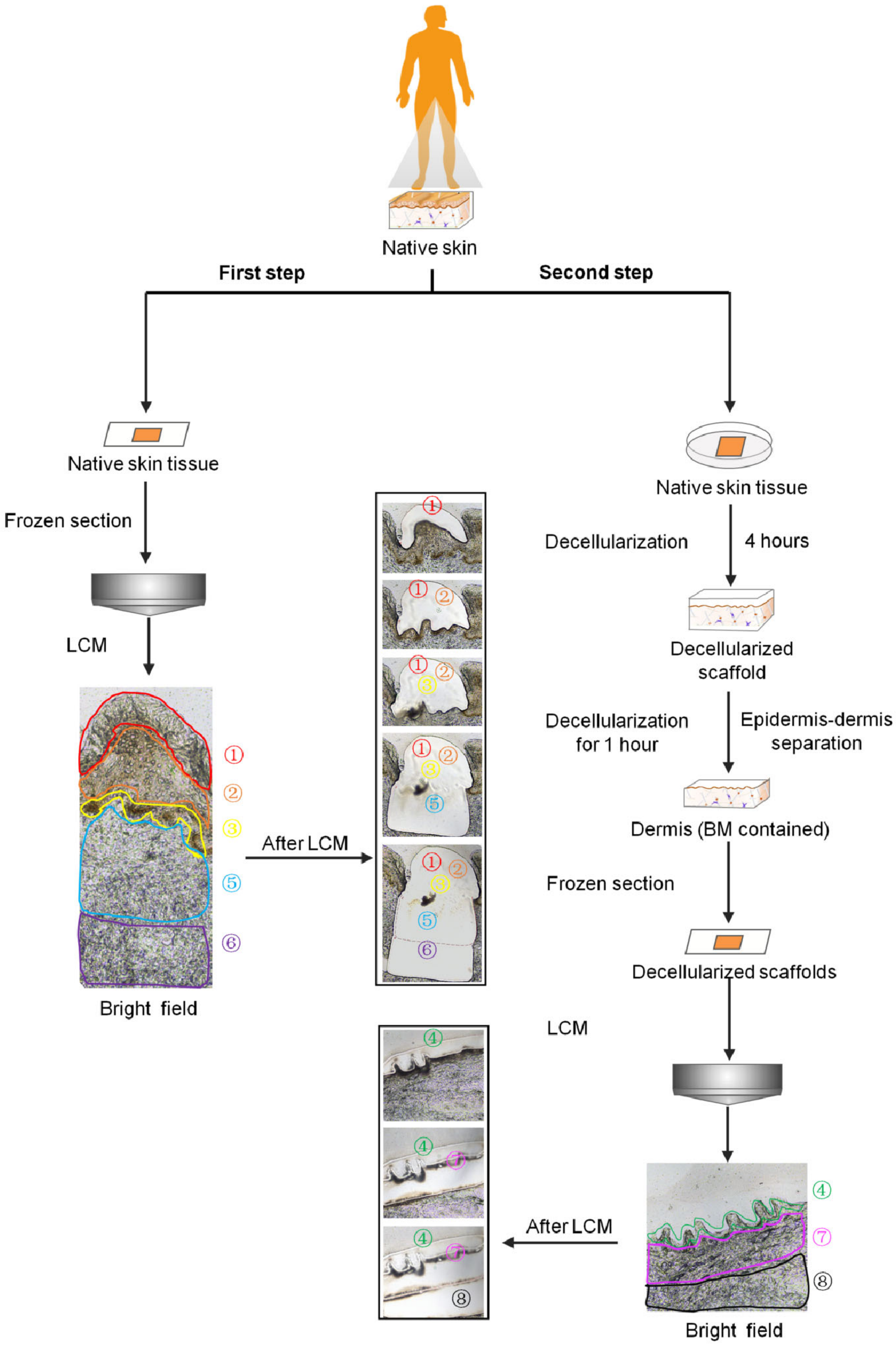

Supplementary Fig. 1 Acquisition of stratified samples. Schematic of experimental workflow. Laser capture microdissection (LCM) was used to dissect, process and analyze five types of native stratified samples and three types of decellularized scaffolds based on skin structure in the study: ① stratum corneum, (SC, red),② granulosum-spinosum (GS, orange), ③ basal layer (BL, yellow), ④ basement membrane (BM, green), ⑤ superficial dermis (SD, blue), ⑥ deep dermis (DD, purple), ⑦ decellularized superficial dermis (decellularized SD, pink), and ⑧ decellularized deep dermis (decellularized DD, black).

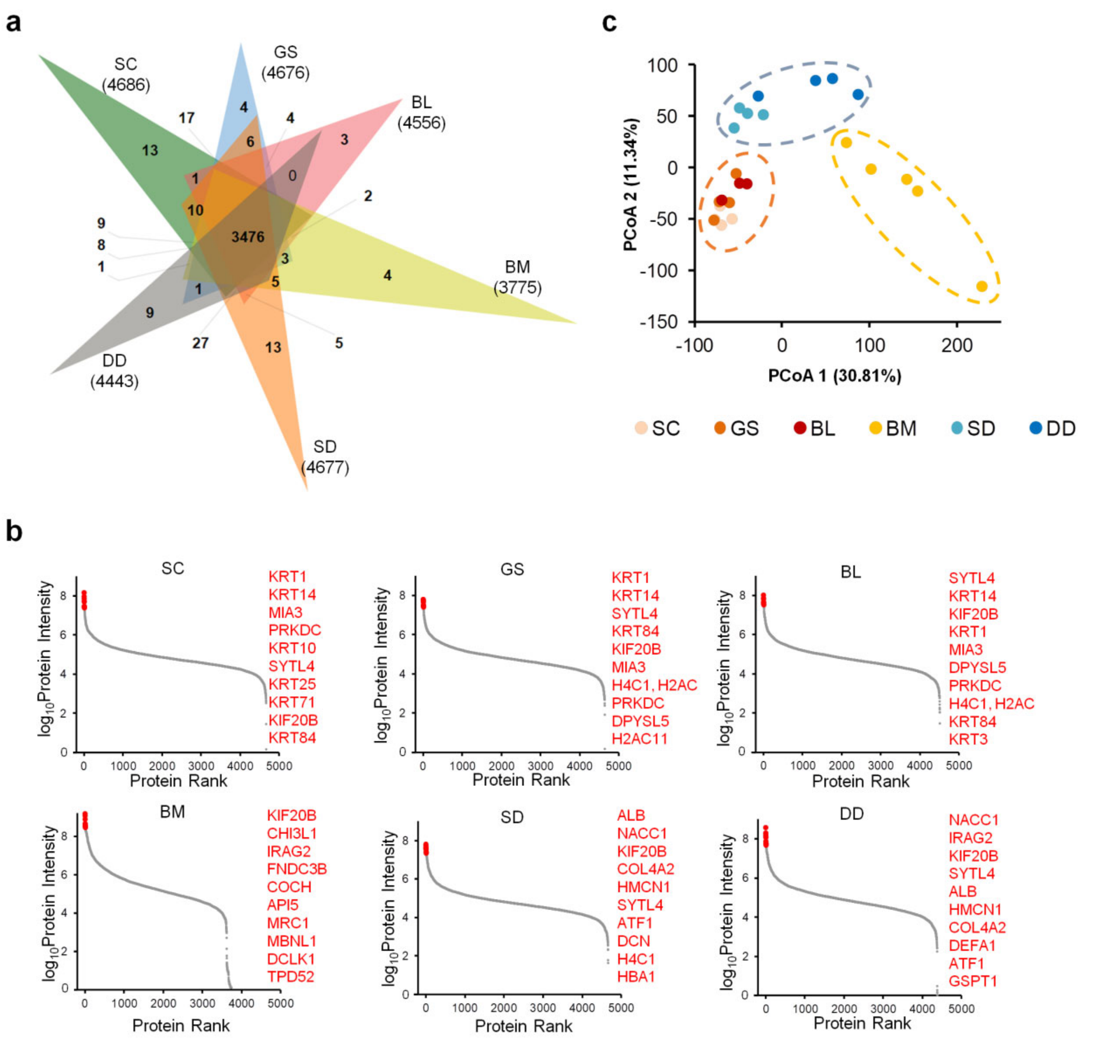

Supplementary Fig. 2 Quantitative proteome profiling of spatially distinct protein signatures in skin tissue. a Overlap among the six layers (SC, GS, BL, BM, SD, and DD) of native stratified skin tissues. **b** Protein intensity distributions of SC, GS, BL, BM, SD, and DD proteomes from native stratified skin tissues. The top ten highly expressed proteins are on the right of the rank diagram. **c** Principal coordinate analysis (PCoA) of the proteome profile of all proteins within six samples based on skin structure. Biological repeats (three to five per sample) produced for each skin sample are represented by different colored points in the figure. Source data are provided as a Source Data file.

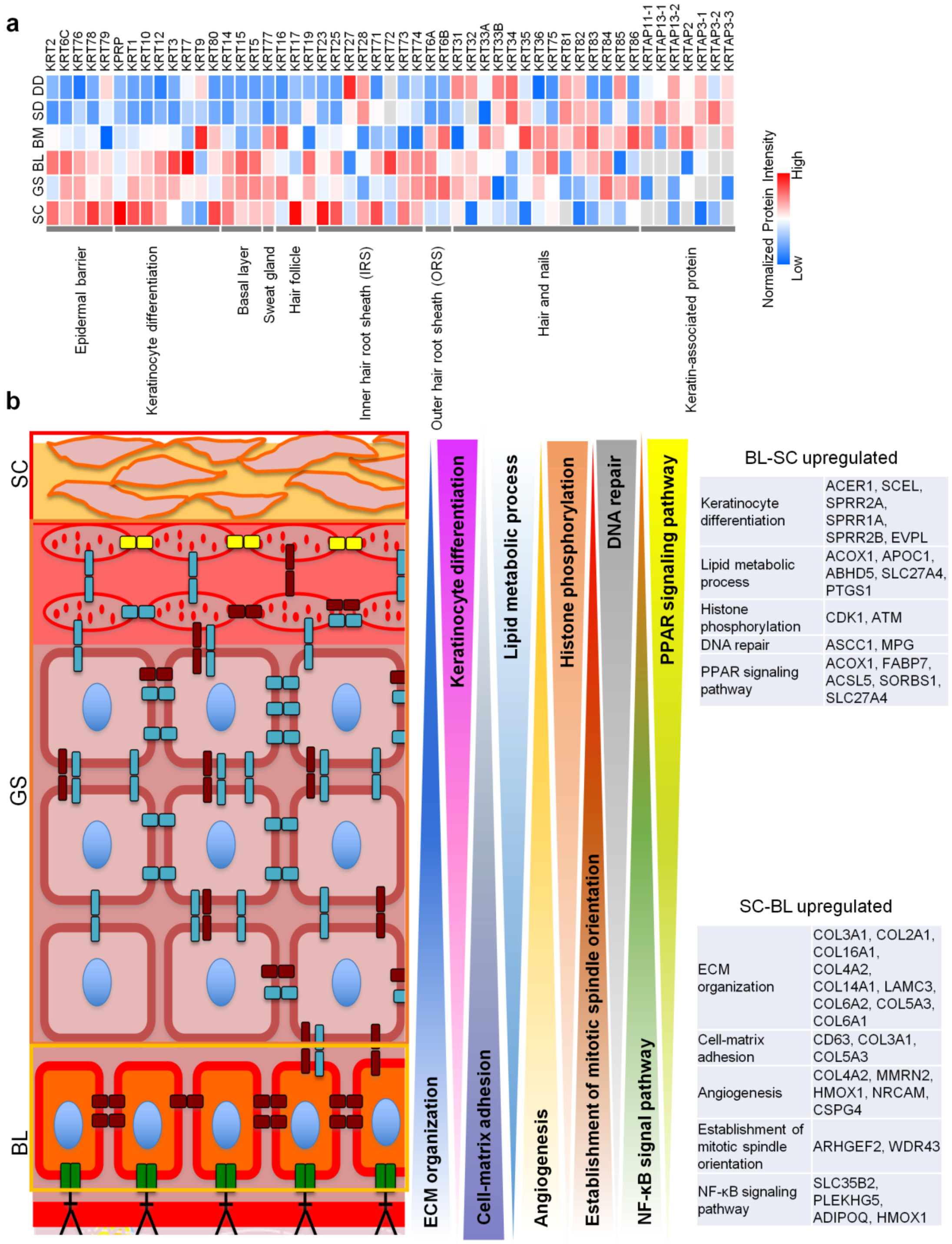

Supplementary Fig. 3 Analysis of functional keratins of stratified native skin. a Functional analysis of the keratins of six layers of native skin tissues. Red and blue boxes indicate the normalized intensity of the enriched or depleted proteins, respectively. **b** Graded distribution of specific biological processes, signals, and the

associated proteins in epidermis from native skin tissues. Source data are provided as a Source Data file.

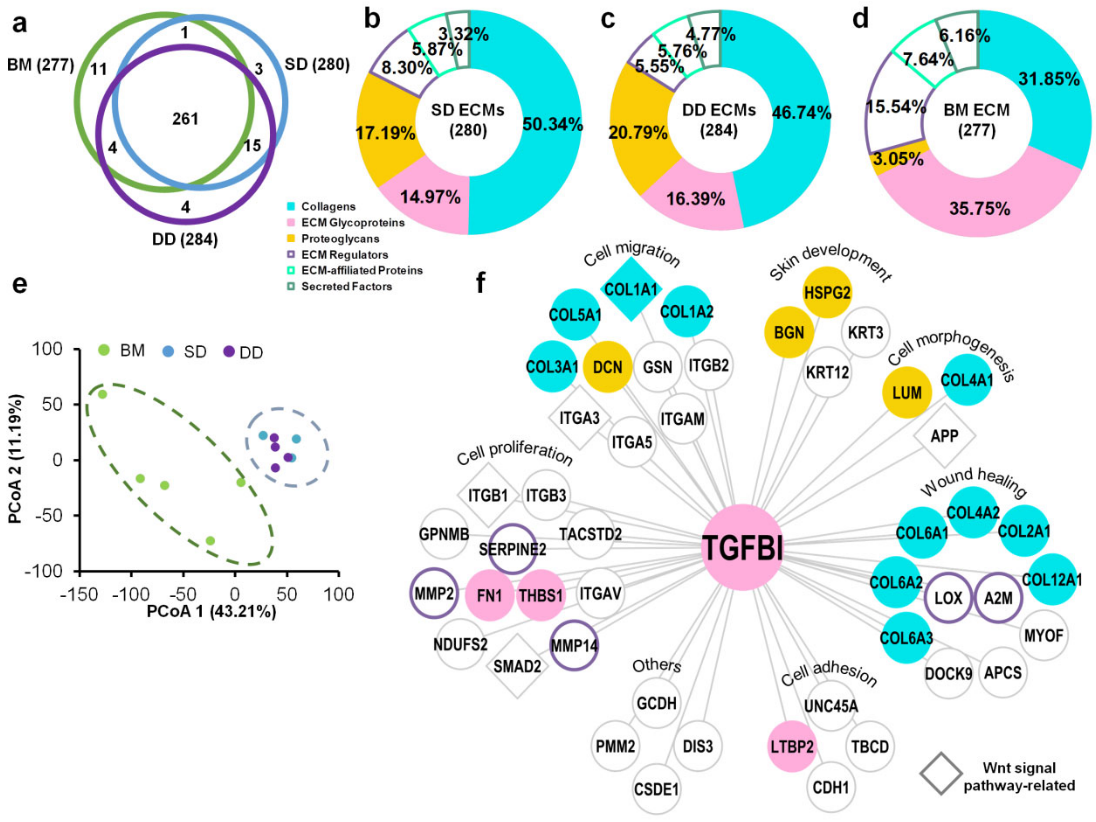

Supplementary Fig. 4 Matrisome analysis of stratified dermis from decellularized scaffolds. a Overlap of the extracellular matrix (ECM) proteins of the scaffolds from the BM, decellularized SD, and decellularized DD samples. **b-d** Pie charts represent the composition ratios of total intensity of six ECM components identified in the BM, decellularized SD, and decellularized DD samples. **e** PCoA of the Matrisome profile of all ECM proteins within three samples, based on the dermis structure. At least three biological repeats were produced for each sample, represented by different color points in the figure. **f** Interactome network of TGFBI and the proteins identified in the basal layer. Gray circles correspond to non-Matrisome components. Different groups represent different functions related to the interacting proteins. The diamond represents the Wnt signal pathway-related proteins. Cyan, pink, and yellow circles, and blue, green, and atrovirens hollow circles correspond to the six components of the skin Matrisome.

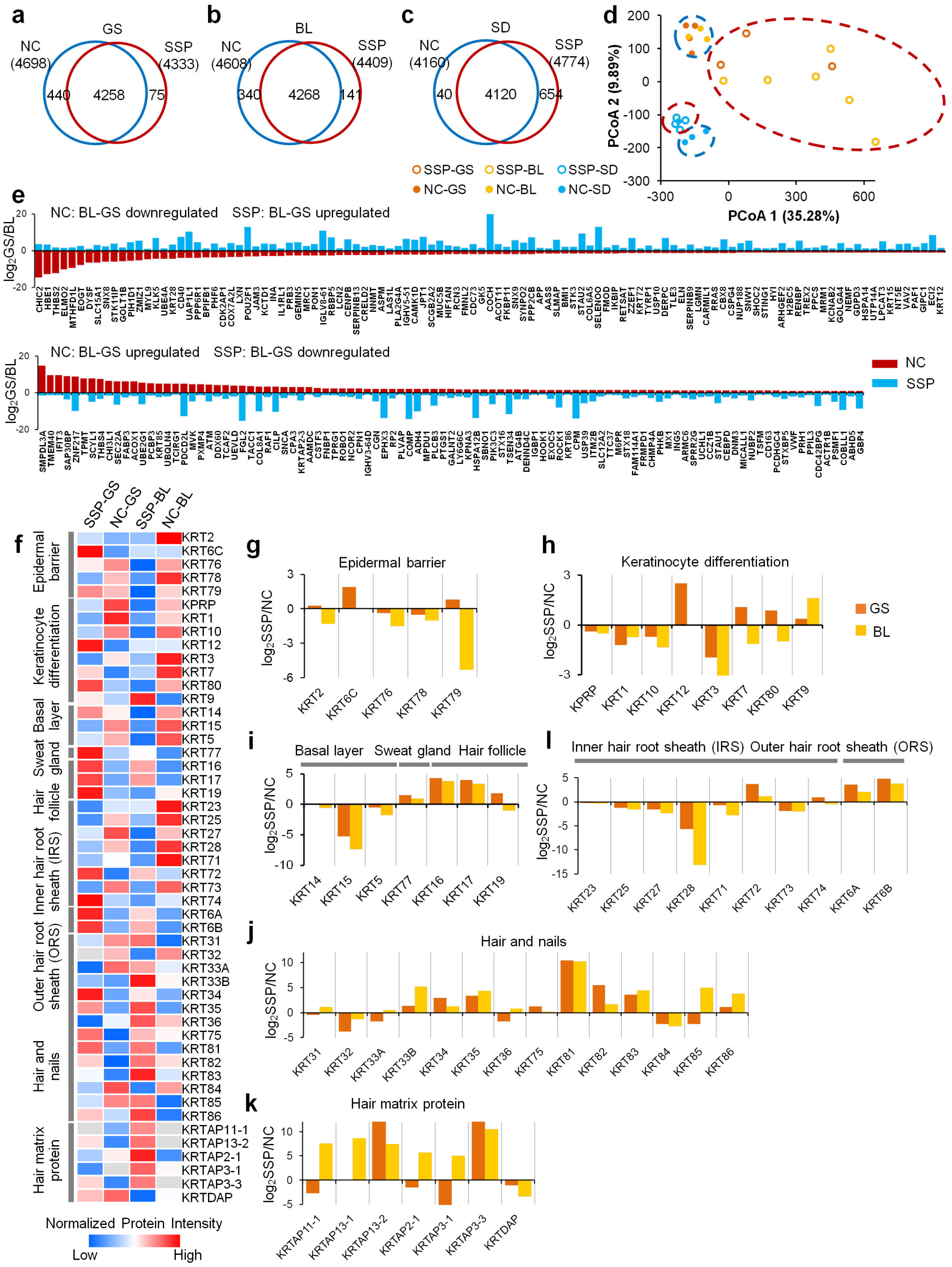

Supplementary Fig. 5 Quantitative proteome profiling of spatially distinct protein signatures in skin tissues from secondary syphilis patients. a-c Overlap of proteins identified in GS, BL, and SD that were coexpressed in secondary syphilis (SSP) and control (NC) groups. **d** PCoA analysis of the proteome profile of GS, BL, and SD in the SSP and control groups. At least three biological repeats were produced

for each sample, represented by different color points in the figure. **e** Histogram analysis of the ratio of the protein intensities from the BL compared to the GS groups. The Y-axis represents the $\log_2$ GS / BL. Red and blue bars represent the control and SSP groups, respectively. **f** Functional analysis of keratin proteins identified in the BL and GS from the SSP and control groups. Red and blue boxes indicate proteins with increased and decreased abundance, respectively. **g-l** Histogram analysis of the ratio of the protein intensities from SSP compared to the control groups. The Y-axis represents the $\log_2$ SSP / Control. Columns colored orange and yellow represent the GS and BL groups. Source data are provided as a Source Data file.

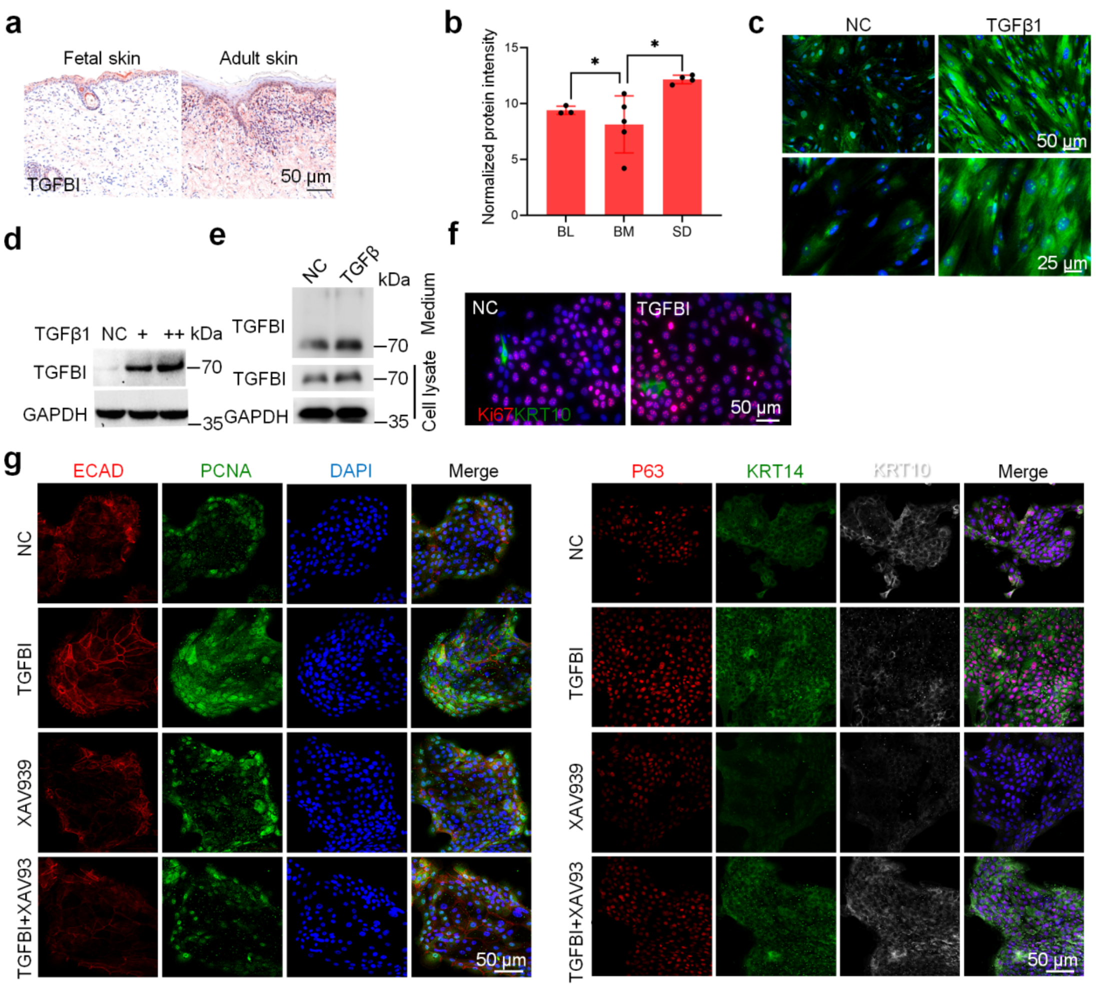

Supplementary Fig. 6. TGFBI enhanced the proliferation of human epidermal stem cells. a Immunohistochemistry of TGFBI in fetal and adult skin tissue (scale bar: 50 μm). **b** Intensities of TGFBI in the layers of BL (n = 3), BM (n = 5), and SD (n = 4) of native skin tissue. Data were show as mean ± SD. Significant difference between BM and other layers was determined by a two tailed t test (*$p < 0.05$, **$p < 0.01$, and ***$p < 0.001$). **c** Immunofluorescence of TGFBI following TGF-β1 treatment of fibroblasts for 24 h (scale bar: 50 and 25 μm). The experiment was repeated three times. **d** TGFBI expression in fibroblasts cultured with TGFβ1 at 2 and 10 ng/ml concentrations for 24 hours. **e** Immunoprecipitation and immunoblotting analysis of TGFBI in cell lysates and supernatant following culture of fibroblasts with TGF-β1 (10 ng/ml) for 24 hours. **f** Ki67 and KRT10 expressions in human EpSCs cultured

with TGFBI at a 100 ng/ml concentration for 48 hours (scale bars: 50 μm). The experiment was repeated three times. **g** Immunofluorescence of ECAD, PCNA, P63, KRT14, and KRT10 on EpSCs with TGFBI and XAV939 treatment for 48 hours culture (scale bars: 50 and 20 μm). The experiment was repeated three times. Source data are provided as a Source Data file.

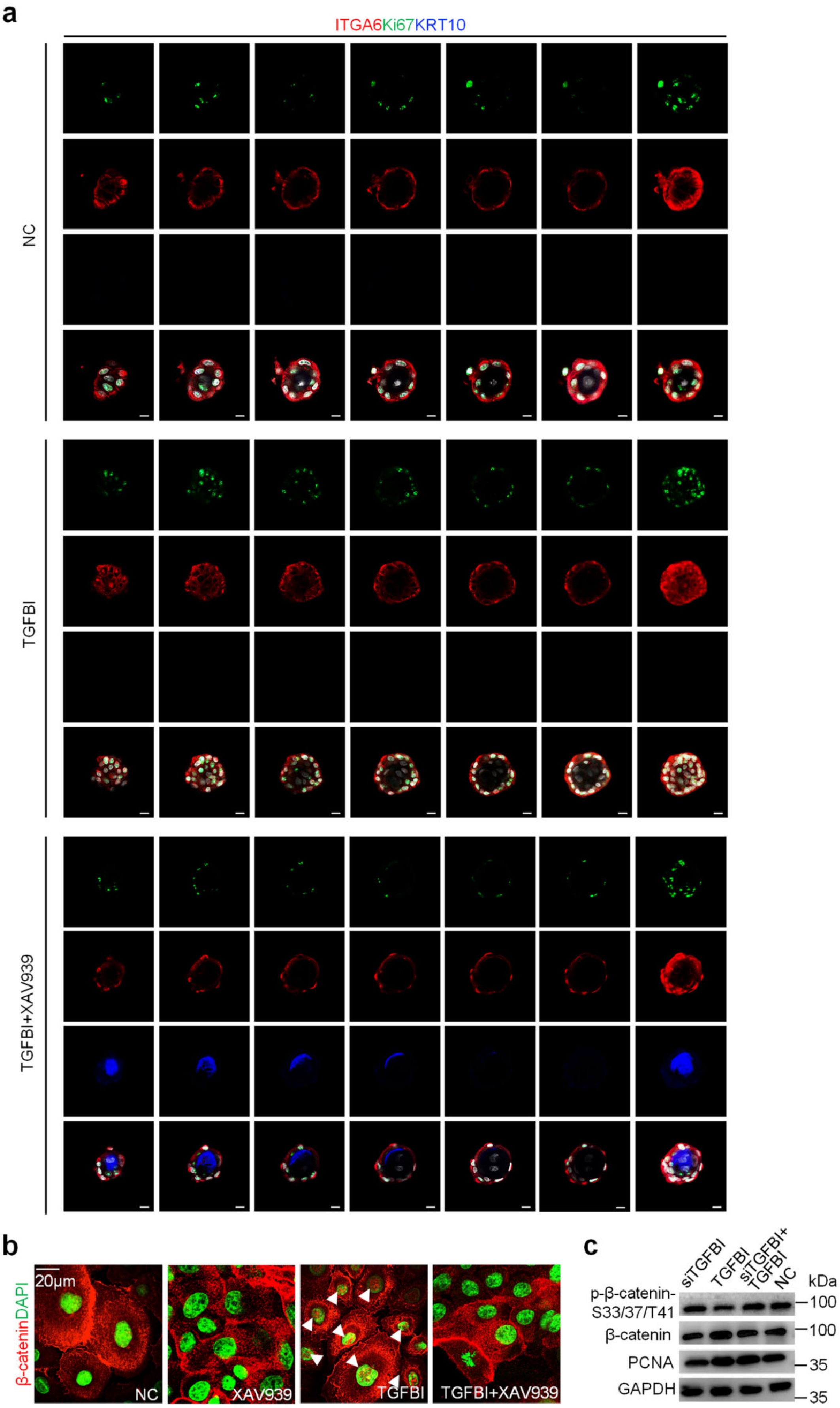

a
ITGA6Ki67KRT10
NC
TGFBI
TGFBI+XAV939
b
β-cateninDAPI
20μm
NC
XAV939
TGFBI
TGFBI+XAV939
c
siTGFBI
TGFBI
siTGFBI+TGFBI
NC
kDa
p-β-catenin-S33/37/T41
100
β-catenin
100
PCNA
35
GAPDH
35

Supplementary Fig. 7 TGFBI enhanced the function of human epidermal stem cells through wnt pathway. a Z-stack immunofluorescence of ITGA6, Ki67 and KRT10 on epidermal organoids treated with TGFBI and XAV939 for seven days of culture (scale bars: 20 μm). The experiment was repeated three times. **b** Immunofluorescence of β-catenin, and DAPI on EpSCs with TGFBI and XAV939 treatment for 48 hours culture (scale bars: 50 and 20 μm). The experiment was repeated three times. **c** Western blot analysis of PCNA, total β-catenin, and p-β-catenin-S33/37/T41 on EpSCs with TGFBI and siTGFBI treatment for 48 hours culture. The experiment was repeated three times.

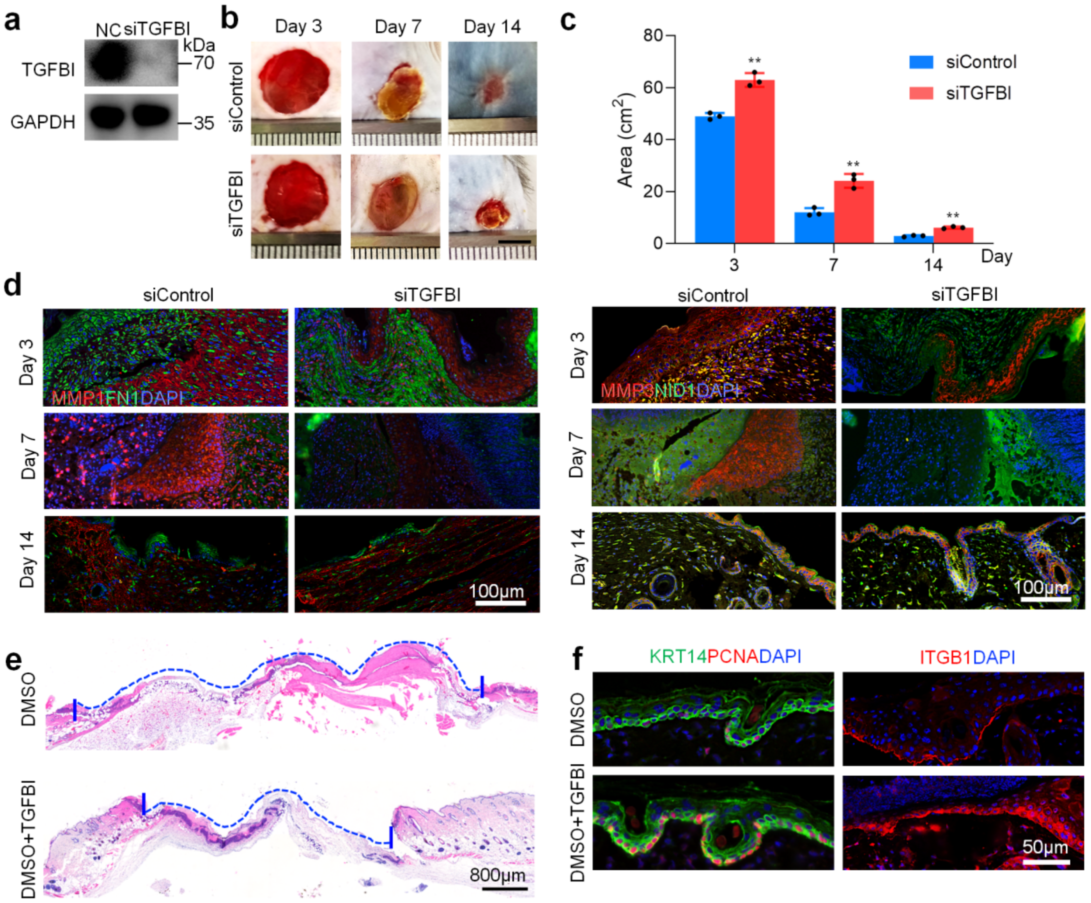

Supplementary Fig. 8 TGFBI promoted cutaneous wound healing through enhancement of re-epithelialization. a Western blot analysis of TGFBI expression on mouse skin tissues with siTGFBI treatment for 48 hours. The experiment was repeated three times. **b** Knockdown of TGFBI during the process of wound healing on mouse skin at day 3, 7, and 14 (n = 3 per group, scale bar: 5 mm). **c** Quantitative analysis of wound area from mouse skin tissues with siTGFBI treatment during wound healing at day 3, 7, and 14 (n = 3 per group). Data were show as mean ± SD. Significant difference between siTGFBI and control group was determined by a two tailed t test (*$p < 0.05$, **$p < 0.01$, and ***$p < 0.001$). **d** Immunofluorescence of MMP1, MMP3, FN1, and NID1 in mouse skin wounds under siTGFBI or siControl treatment at 0, 3, 7, or 14 days (scale bars: 800, 100 and 50 μm). The experiment was repeated three times. **e** Hematoxylin & Eosin (HE) staining in mouse skin wounds

with or without TGFBI treatment for 3 days (scale bars: 800 μm). The experiment was repeated three times. The blue dotted lines indicate the unhealed wound sites of the mice. **f** Immunofluorescence of KRT14, PCNA, and ITGB1 in mouse skin wounds at day 7 (scale bars: 50 μm). The experiment was repeated three times. Source data are provided as a Source Data file.

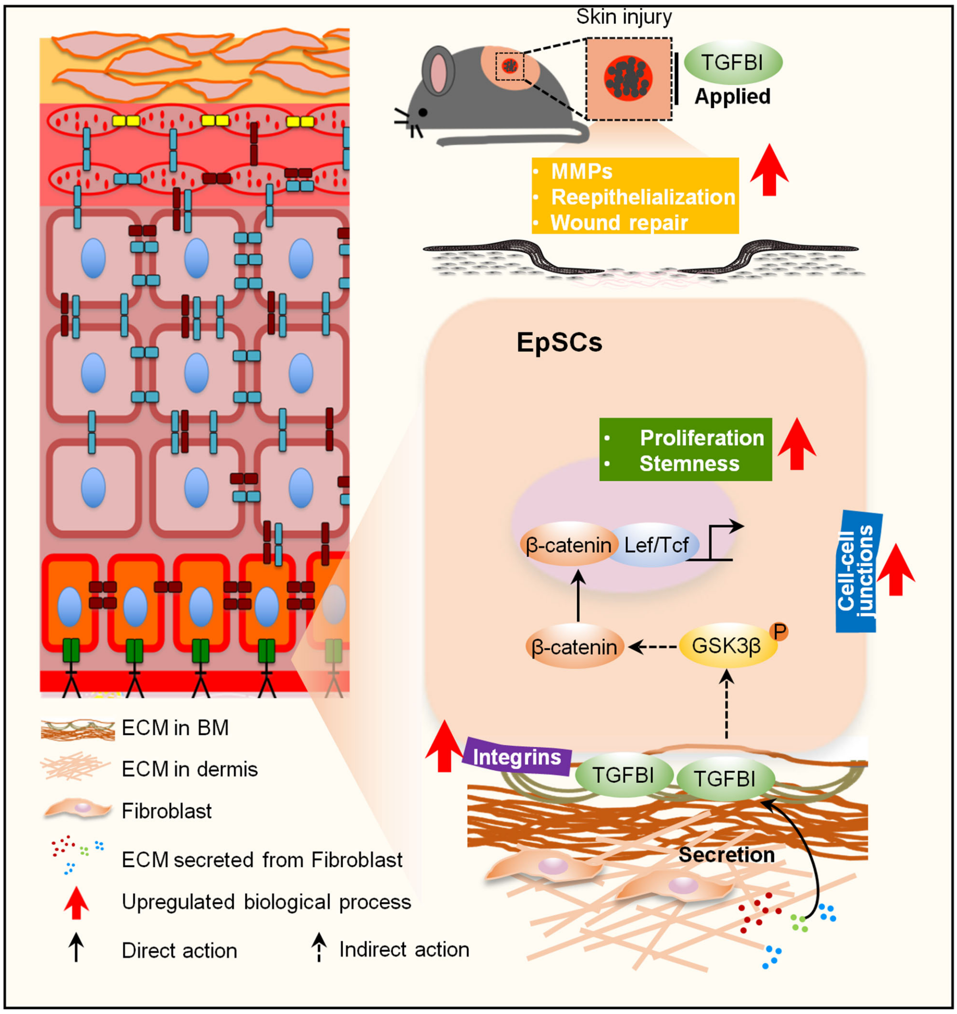

Supplementary Fig. 9 Schematic diagram of TGFBI promoting skin re-epidermization during wound healing through enhancing the growth of EpSCs. Fibroblasts in the dermis secrete ECM protein TGFBI, located in the BM. Then, TGFBI promotes the proliferation and stemness of EpSCs by activating the Wnt pathway. In addition, TGFBI can also promote the expression level of integrin and cell-cell junction-associated proteins, enhancing epithelialization. Further, damaged skin tissues of mice were treated with TGFBI, which can promote the expression of MMPs during wound healing and accelerate the process of cell migration and re-epidermization, improving the efficiency of wound repair.

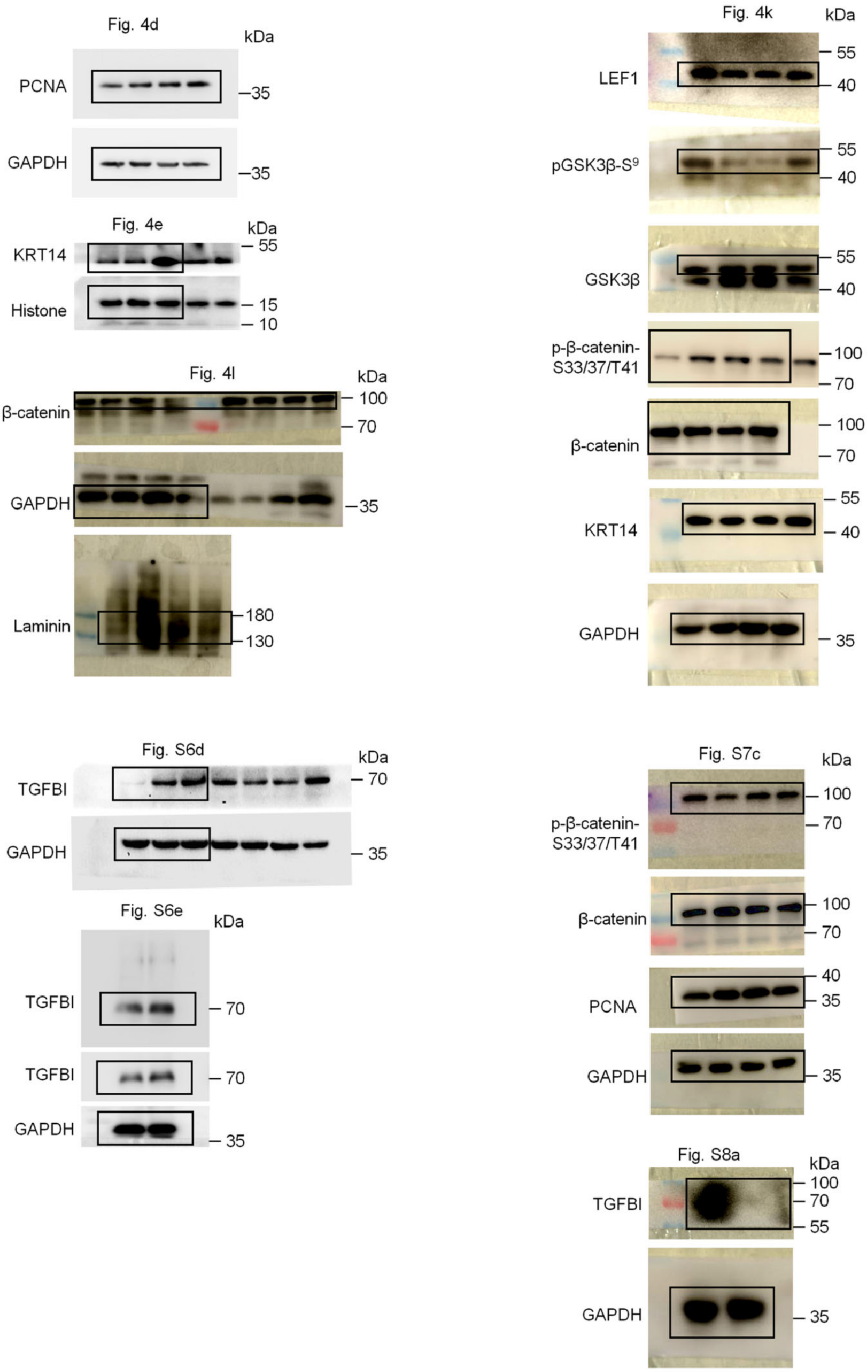

Supplementary Fig. 10 Uncropped scans of Western blots.
