# Supplementary material for: Spatially resolved proteomic map shows that extracellular matrix regulates epidermal growth
Source: Nat Commun. 2022 Jul 11;13:4012. doi: 10.1038/s41467-022-31659-9 (PMC9273758; doi:10.1038/s41467-022-31659-9)
Supplement: Supplementary file 3 — Description of Additional Supplementary Files [file 41467_2022_31659_MOESM3_ESM.pdf]

Description of Additional Supplementary Files

File Name: Supplementary Data 1
Description: Overview of the characteristics of patients diagnosed with Syphilis involved in the study.

File Name: Supplementary Data 2
Description: All protein identified in six different skin layers of stratum corneum (SC), granular-spinous (GS), basal layer (BL), Basement membrane (BM), superficial dermis (SD), and deep dermis (DD).

File Name: Supplementary Data 3
Description: All ECM proteins identified in decellularization skin layers of basement membrane (BM), superficial dermis (SD), and deep dermis (DD).

File Name: Supplementary Data 4
Description: All protein identified in different skin layers of secondary syphilis (SSP) patients.

File Name: Supplementary Data 5
Description: Differentially expressed genes in TGFBI treated and untreated samples.

File Name: Supplementary Data 6
Description: Differentially expressed proteins of SSP-EpSCs with or without TGFBI treatment in the control and SSP groups.

File Name: Supplementary Data 7
Description: Details of primer sequences used for PCR in this study.

File Name: Supplementary Data 8
Description: Companies providing equipment, reagents and/or supplies.

File Name: Supplementary Movie 1
Description: Procedure of obtaining stratum corneum (SC) using laser capture microdissection (LCM).

File Name: Supplementary Movie 2
Description: Procedure of obtaining granulosum-spinosum (GS) using LCM.

File Name: Supplementary Movie 3
Description: Procedure of obtaining basal layer (BL) using LCM.

File Name: Supplementary Movie 4
Description: Procedure of obtaining surperficial dermis (SD) using LCM.

File Name: Supplementary Movie 5
Description: Procedure of obtaining deep dermis (DD) using LCM.

File Name: Supplementary Movie 6
Description: Procedure of obtaining decellularization skin layer of basement membrane (BM) using LCM.

File Name: Supplementary Movie 7
Description: Procedure of obtaining decellularized SD using LCM.

File Name: Supplementary Movie 8
Description: Procedure of obtaining decellularized DD using LCM.
