# Supplementary material for: Spatially resolved proteomic map shows that extracellular matrix regulates epidermal growth
Source: Nat Commun. 2022 Jul 11;13:4012. doi: 10.1038/s41467-022-31659-9 (PMC9273758; doi:10.1038/s41467-022-31659-9)
Supplement: Supplementary file 20 — Reporting Summary [file 41467_2022_31659_MOESM20_ESM.pdf]

nature portfolio

Corresponding author(s): Ling Leng, Jun Li, Yunping Zhu

Last updated by author(s): May 26, 2022

Reporting Summary

Nature Portfolio wishes to improve the reproducibility of the work that we publish. This form provides structure for consistency and transparency in reporting. For further information on Nature Portfolio policies, see our Editorial Policies and the Editorial Policy Checklist.

Statistics

For all statistical analyses, confirm that the following items are present in the figure legend, table legend, main text, or Methods section.

n/a	Confirmed	
☐	☒	The exact sample size (n) for each experimental group/condition, given as a discrete number and unit of measurement
☐	☒	A statement on whether measurements were taken from distinct samples or whether the same sample was measured repeatedly
☐	☒	The statistical test(s) used AND whether they are one- or two-sided<br>*Only common tests should be described solely by name; describe more complex techniques in the Methods section.*
☒	☐	A description of all covariates tested
☐	☒	A description of any assumptions or corrections, such as tests of normality and adjustment for multiple comparisons
☐	☒	A full description of the statistical parameters including central tendency (e.g. means) or other basic estimates (e.g. regression coefficient) AND variation (e.g. standard deviation) or associated estimates of uncertainty (e.g. confidence intervals)
☐	☒	For null hypothesis testing, the test statistic (e.g. F, t, r) with confidence intervals, effect sizes, degrees of freedom and P value noted<br>*Give P values as exact values whenever suitable.*
☒	☐	For Bayesian analysis, information on the choice of priors and Markov chain Monte Carlo settings
☐	☒	For hierarchical and complex designs, identification of the appropriate level for tests and full reporting of outcomes
☒	☐	Estimates of effect sizes (e.g. Cohen's d, Pearson's r), indicating how they were calculated

Our web collection on statistics for biologists contains articles on many of the points above.

Software and code

Policy information about availability of computer code

Data collection	Mass spectrometry datasets were collected using a Q Exactive HF-X Orbitrap mass spectrometer (Thermo Fisher Scientific) coupled with an EASY-nLC 1200 (Thermo Fisher Scientific). The data-independent acquisition (DIA) scan mode was used for single-shot samples. The fractionated samples of the pool were acquired using the top 40 data-dependent acquisition (DDA) scan mode. The MS was operated using Xcalibur software (version 4.1).
Data analysis	The DDA data was analyzed by Proteome Discoverer (version 2.4) software and was used to generate a spectral library in Spectronaut (version 14.9.201124.47784). The DIA data were processed on Spectronaut (version 14.9.201124.47784) using the default settings.<br>General data analysis including the normalization and transformation was performed using the R package software (version 4.0.3). Differential analysis of the processed DIA data was performed using the R package Limma (version 3.46.0). The statistical analysis for other experiment results was performed using GraphPad Prism (version 9.0.0). Principal coordinates analysis was performed using the R package ape (version 5.4.1). The heatmaps of protein quantitation values were displayed using Perseus software (version 1.6.0.7). The protein interactome network was constructed using Cytoscape (version 3.8.2). The ImageJ software (version 1.53) was used to detect and quantify the fluorescence intensities of protein expressions.

For manuscripts utilizing custom algorithms or software that are central to the research but not yet described in published literature, software must be made available to editors and reviewers. We strongly encourage code deposition in a community repository (e.g. GitHub). See the Nature Portfolio guidelines for submitting code & software for further information.

Data

Policy information about availability of data

All manuscripts must include a data availability statement. This statement should provide the following information, where applicable:

- Accession codes, unique identifiers, or web links for publicly available datasets
- A description of any restrictions on data availability
- For clinical datasets or third party data, please ensure that the statement adheres to our policy

All proteomics raw data have been deposited to the ProteomeXchange Consortium via the iProX partner repository with the dataset identifier PXD027093. UniProt reference proteome sequences of "Homo sapiens" with 20,279 entries was downloaded from in February 2021 (https://www.uniprot.org/downloads). The Gene Ontology annotations of biological processes and cellular components for proteins were obtained from the online resource DAVID (https://david.ncifcrf.gov, 2021 Update). The biological pathway annotations were obtained from KEGG pathway database (https://www.kegg.jp/kegg/pathway.html). The protein-protein interactions were retrieved from the STRING database (https://string-db.org/). The extracellular matrix protein annotations were obtained from the Matrisome database (http://matrisomeproject.mit.edu/proteins/).

Field-specific reporting

Please select the one below that is the best fit for your research. If you are not sure, read the appropriate sections before making your selection.

☒ Life sciences ☐ Behavioural & social sciences ☐ Ecological, evolutionary & environmental sciences

For a reference copy of the document with all sections, see nature.com/documents/nr-reporting-summary-flat.pdf

Life sciences study design

All studies must disclose on these points even when the disclosure is negative.

Sample size	No sample size calculation was performed. Five patients who were diagnosed with secondary syphilis were included. Also, five skin tissues from donors without syphilis are used as control groups. Six different skin layers of stratum corneum (SC), granular-spinous (GS), basal layer (BL), Basement membrane (BM), superficial dermis (SD), and deep dermis (DD) were obtained using laser capture microdissection (LCM) from the control groups, and Six different skin layers of SC, BL, and SD were obtained using LCM from the second syphilis patients. For each skin layer, at least three biological repeats were performed ($n \geq 3$), with most cases of n =4 or 5. It can provided enough statistical power when comparing protein expresses among different replicates. Also, the multiple tests (Benjamini-Hochberg adjust) and analyses were performed as described in the manuscript to ensure the samples are representative and results are conclusive. For other experiments, including western blot, QPCR, immunofluorescence, mouse wound healing experiment and so on, each was performed at least three times ($n \geq 3$). This number is sufficient for robust statistical analyses.
Data exclusions	No data has been excluded.
Replication	For all experiments, including the MS experiment and verification experiment, at least three repeats ($n \geq 3$) were performed. The number of replicates for each specific experiment is indicated in the main text, figure legends, and methods. All attempts of replication were successful. To verify the reproducibility of MS platform, the HEK293T cell lysate was measured every two days as the quality-control standard sample. The HEK293T standard sample was digested and analyzed using the same method, condition, and MS instrument as the skin samples. In the whole procedure of MS experiment, six HEK293T standard samples were analyzed and six MS datasets were generated. A pairwise spearman correlation coefficient was calculated for protein quantification of all quality-control samples. The average correlation coefficient among the standards was 0.98, indicating the reproducibility of the MS platform. The details has been described in the methods section.
Randomization	This study includes two groups, the secondary syphilis (SSP) patients group and control donors group. The patients and the matched controls that conformed to the inclusion criteria were randomly sampled. The control donors were anonymous. The samples in two groups were prepared for MS analysis using exactly the same procedures, and then the peptide mixtures of each sample were measured by MS in randomized order. For the mouse wound healing experiment, the mice were allocated to TFGBI-treatment or control group randomizedly. For other validation experiments of samples from SSP patients and control individuals, the measurements were randomized in groups.
Blinding	The tissue samples preparation and peptide mixtures analysis by mass spectrometer were processed by two different investigators. Both investigators were kept blinded to the group (control or secondary syphilis) and layer (skin layers) information. All samples were analyzed using exactly the same procedures. For other experiments, each sample was assigned with a number without indication of the group (e.g. LL_01, LL_02, ...), the investigators who performed the experiments and collected the data were kept blinded to the group allocation information. When all data collection finished, the investigators who were responsible for data analysis were informed about the group allocation information.

Reporting for specific materials, systems and methods

We require information from authors about some types of materials, experimental systems and methods used in many studies. Here, indicate whether each material, system or method listed is relevant to your study. If you are not sure if a list item applies to your research, read the appropriate section before selecting a response.

Materials & experimental systems

n/a	Involved in the study	
☐	☒	Antibodies
☐	☒	Eukaryotic cell lines
☒	☐	Palaeontology and archaeology
☐	☒	Animals and other organisms
☐	☒	Human research participants
☒	☐	Clinical data
☒	☐	Dual use research of concern

Methods

n/a	Involved in the study	
☒	☐	ChIP-seq
☒	☐	Flow cytometry
☒	☐	MRI-based neuroimaging

Antibodies

Antibodies used

IHC/IF antibodies:
Treponema pallidum (Novus Biologicals NB110-17242, 1:100), Collagen IV (Proteintech 55131-1-AP, 1:1000), Collagen XVII (Abcam ab184996, clone EPR18614, 1:100), Collagen VII (Santa cruz biotechnology sc-33710, clone 4D2, 1:50), Cytokeratin 1 (Abcam ab93652, 1:200), Cytokeratin 10 (Abcam ab9026, clone DE-K10, 1:100), Cytokeratin 14 (Abcam ab181595, clone EPR17350, 1:1000), Cytokeratin 14 (Abcam ab7800, clone LL002, 1:400), DSG1 (Proteintech 24587-1-AP, 1:50), DSG1 (Invitrogen MA1-91590, clone 3G131, 1:50), DSG2 (Invitrogen 14-9159-82, clone CSTEM28, 1:50), Claudin 1 (Abcam ab211737, clone EPRR18871, 1:1000), EGFR (Cell Signaling Technology 4267S, clone D38B1, 1:50), ITGA6 (Abcam ab20142, clone MP 4F10, 1:500), ITGB1(Abcam ab78502, clone P4G11, 1:100), Laminin (Abcam ab11575, 1:50), p63 (Abcam ab735, clone 4A4, 1:200), Ki67 (Abcam ab15580, 1:200), Plectin (Santa cruz biotechnology sc-33649, clone 10F6, 1:50), TGFBI (Abcam ab170874, clone EPR12078(B), 1:100), TGFBI (Proteintech 10188-1-AP, 1:200), PCNA (Cell Signaling Technology 2586S, clone PC10, 1:2400), E-cadherin (Proteintech 20874-1-AP, 1:200), β-Catenin (Cell Signaling Technology 8480S, clone D10A8, 1:100), MMP1 (Proteintech 10371-2-AP, 1:100), MMP3 (Proteintech 66338-1-Ig, clone 1F5A9, 1:1000), MMP7 (Proteintech 10374-2-AP, 1:200), Nidogen 1 (Proteintech 13766-1-AP, 1:200), Fibronectin (Abcam ab2413, 1:100), Alexa Fluor® 488 Goat anti-mouse IgG (Invitrogen A32723, 1:500), Alexa Fluor® 488 Goat anti-mouse IgG2a (Invitrogen A21131, 1:500), Alexa Fluor® 488 Goat anti-rabbit IgG (Invitrogen A11008, 1:500), Alexa Fluor® 568 Goat anti-mouse IgG1 (Invitrogen A21124, 1:500), Alexa Fluor® 647 Goat anti-mouse IgG1 (Invitrogen A21240, 1:500), Alexa Fluor® 568 Goat anti-mouse IgG2a (Invitrogen A21134, 1:500), Alexa Fluor® 568 Goat anti-rabbit IgG (Invitrogen A11011, 1:500), Alexa Fluor® 647 Goat anti-mouse IgG2b (Invitrogen A21242, 1:500), Alexa Fluor® 488 Goat anti-mouse IgG3 (Invitrogen A21151, 1:500).
WB antibodies:
Histone (GeneTex GTX122148, 1:3000), Cytokeratin 14 (Abcam ab181595, clone EPR17350, 1:20000), LEF1 (Cell Signaling Technology 2230S, clone C12A5, 1:1000), GSK3β (Bioworld BS6886, 1:1000), Phospho-GSK-3β (Cell Signaling Technology 5558S, clone D85E12, 1:1000), β-catenin (Cell Signaling Technology 8480S, clone D10A8, 1:1000), p-β-catenin(S33/37/T41) (Cell Signaling Technology 9561S, 1:1000), GAPDH (Proteintech HRP-60004, clone 1E6D9, 1:10000), GAPAH (Cell Signaling Technology 8884S, clone D16H11, 1:1000), TGFBI (Cell Signaling Technology 5601S, clone D31B8, 1:1000), Laminin (Abcam ab78286, clone P3H9-2, 1:500), PCNA (Cell Signaling Technology 2586S, clone PC10, 1:1000).

Validation

IHC/IF antibodies verified by supplier:
Treponema pallidum (Novus Biologicals NB110-17242)
https://www.citeab.com/antibodies/451693-nb110-17242-treponema-pallidum-antibody?des=97fee39a4a57de16
Collagen IV (Proteintech 55131-1-AP)
https://www.citeab.com/antibodies/2185269-55131-1-ap-collagen-type-iv-antibody?des=4778889dae2bf808
Collagen XVII (Abcam ab184996, clone EPR18614)
https://www.citeab.com/antibodies/2928459-ab184996-anti-collagen-xvii-antibody-epr18614?des=5413b63d87b44541
Collagen VII (Santa cruz biotechnology sc-33710, clone 4D2)
https://www.citeab.com/antibodies/783010-sc-33710-anti-col7a1-antibody-4d2?des=80793ff5ecfd0bfd
Cytokeratin 1 (Abcam ab93652)
https://www.citeab.com/antibodies/726131-ab93652-anti-cytokeratin-1-antibody?des=9eea4b8034fef8a7
Cytokeratin 10 (Abcam ab9026, clone DE-K10)
https://www.citeab.com/antibodies/726144-ab9026-anti-cytokeratin-10-antibody-de-k10-cytosk?des=5c7734694ea08e99l
Cytokeratin 14 (Abcam ab181595, clone EPR17350)
https://www.citeab.com/antibodies/2435539-ab181595-anti-cytokeratin-14-antibody-epr17350-c?des=cf75cb422bd47026
Cytokeratin 14 (Abcam ab7800, clone LL002)
https://www.citeab.com/antibodies/726175-ab7800-anti-cytokeratin-14-antibody-ll002?des=ca2a86c50d0640cd
DSG1 (Proteintech 24587-1-AP)
https://www.citeab.com/antibodies/2824796-24587-1-ap-dsg1-antibody?des=9c48944e4e8b73f3
DSG1 (Invitrogen MA1-91590, clone 3G131)
https://www.citeab.com/antibodies/89588-ma1-91590-desmoglein-1-monoclonal-antibody-3g131?des=10f7ea440e4be940
DSG2 (Invitrogen 14-9159-82, clone CSTEM28)
https://www.citeab.com/antibodies/4059679-14-9159-82-desmoglein-2-monoclonal-antibody-cstem28?des=0c5b9e5d95d69b79
Claudin 1 (Abcam ab211737, clone EPRR18871)
https://www.citeab.com/antibodies/4636707-ab211737-anti-claudin-1-antibody-eprr18871?des=8fa0176b22a97e5d
EGFR (Cell Signaling Technology 4267S, clone D38B1)
https://www.citeab.com/antibodies/124119-4267-egf-receptor-d38b1-xp-rabbit-mab?des=eef270307377f7b2
ITGA6 (Abcam ab20142, clone MP 4F10)
https://www.citeab.com/antibodies/767679-ab20142-anti-integrin-alpha-6-antibody-mp-4f10?des=62081e535748c7f7
ITGB1(Abcam ab78502, clone P4G11)
https://www.citeab.com/antibodies/767937-ab78502-anti-integrin-beta-1-antibody-p4g11?des=af11d96d5c1d453c
Laminin (Abcam ab11575)
https://www.citeab.com/antibodies/741590-ab11575-anti-laminin-antibody?des=eb58e28c0349a06b

p63 (Abcam ab735, clone 4A4)
https://www.citeab.com/antibodies/1899477-ab735-anti-p63-antibody-4a4?des=46a051ebdd4d2bde
Ki67 (Abcam ab15580)
https://www.citeab.com/antibodies/1896848-ab15580-anti-ki67-antibody?des=8857c778d881a303
Plectin (Santa cruz biotechnology sc-33649, clone 10F6)
https://www.citeab.com/antibodies/823656-sc-33649-anti-plectin-antibody-10f6?des=b4b6f0699f37debb
TGFBI (Abcam ab170874, clone EPR12078(B))
https://www.citeab.com/antibodies/1903059-ab170874-anti-tgfbi-antibody-epr12078-b?des=5f0f02a50e23b347
TGFBI (Proteintech 10188-1-AP)
https://www.citeab.com/antibodies/80958-10188-1-ap-tgfbi-bigh3-antibody?des=55a6d1c1d205cb8f
PCNA (Cell Signaling Technology 2586S, clone PC10)
https://www.citeab.com/antibodies/123622-2586-pcna-pc10-mouse-mab?des=cdf34798de098a3c
E-cadherin (Proteintech 20874-1-AP)
https://www.citeab.com/antibodies/575608-20874-1-ap-e-cadherin-antibody?des=585313f140141d23
β-Catenin (Cell Signaling Technology 8480S, clone D10A8)
https://www.citeab.com/antibodies/125282-8480-catenin-d10a8-xp-rabbit-mab?des=fb5c735d6656467e
MMP1 (Proteintech 10371-2-AP)
https://www.citeab.com/antibodies/81256-10371-2-ap-mmp1-antibody?des=bb9a5139396c23b5
MMP3 (Proteintech 66338-1-Ig, clone 1F5A9)
https://www.citeab.com/antibodies/3343896-66338-1-ig-mmp3-antibody?des=b3a194318bc7a45b
MMP7 (Proteintech 10374-2-AP)
https://www.citeab.com/antibodies/81262-10374-2-ap-mmp7-antibody?des=22936fb1b8806686
Nidogen 1 (Proteintech 13766-1-AP)
https://www.citeab.com/antibodies/977850-13766-1-ap-entactin-antibody?des=a71e7dc5fdab81d4
Fibronectin (Abcam ab2413, 1:100)
https://www.citeab.com/antibodies/730636-ab2413-anti-fibronectin-antibody?des=31398c9b23f69a1e
Alexa Fluor® 488 Goat anti-mouse IgG (Invitrogen A32723)
https://www.citeab.com/antibodies/4069879-a32723-goat-anti-mouse-igg-h-l-highly-cross-adsorb?des=5aeb4dceb3c7e5cc
Alexa Fluor® 488 Goat anti-mouse IgG2a (Invitrogen A21131)
https://www.citeab.com/antibodies/2401235-a-21131-goat-anti-mouse-igg2a-cross-adsorbed-seconda?des=dc1922d845ff0b34
Alexa Fluor® 488 Goat anti-rabbit IgG (Invitrogen A11008)
https://www.citeab.com/antibodies/2400604-a-11008-goat-anti-rabbit-igg-h-l-cross-adsorbed-se?des=63fa950fb60d6ac6
Alexa Fluor® 568 Goat anti-mouse IgG1 (Invitrogen A21124)
https://www.citeab.com/antibodies/2401230-a-21124-goat-anti-mouse-igg1-cross-adsorbed-secondar?des=9b48429f8d3ac9a6
Alexa Fluor® 647 Goat anti-mouse IgG1 (Invitrogen A21240)
https://www.citeab.com/antibodies/2401273-a-21240-goat-anti-mouse-igg1-cross-adsorbed-secondar?des=1a00f42734cb22ad
Alexa Fluor® 568 Goat anti-mouse IgG2a (Invitrogen A21134)
https://www.citeab.com/antibodies/2401237-a-21134-goat-anti-mouse-igg2a-cross-adsorbed-seconda?des=ad00a3c6acc5e792
Alexa Fluor® 568 Goat anti-rabbit IgG (Invitrogen A11011)
https://www.citeab.com/antibodies/2400607-a-11011-goat-anti-rabbit-igg-h-l-cross-adsorbed-se?des=13dfb8697d0f0d33
Alexa Fluor® 647 Goat anti-mouse IgG2b (Invitrogen A21242)
https://www.citeab.com/antibodies/2401275-a-21242-goat-anti-mouse-igg2b-cross-adsorbed-seconda?des=f9a8690aa1e0a962
Alexa Fluor® 488 Goat anti-mouse IgG3 (Invitrogen A21151)
https://www.citeab.com/antibodies/2401248-a-21151-goat-anti-mouse-igg3-cross-adsorbed-secondar?des=d184993811f337f6
WB antibodies verified by supplier:
Histone (GeneTex GTX122148)
https://www.citeab.com/antibodies/553423-gtx122148-histone-h3-antibody?des=0dc65b047ae56a09
Cytokeratin 14 (Abcam ab181595, clone EPR17350)
https://www.citeab.com/antibodies/2435539-ab181595-anti-cytokeratin-14-antibody-epr17350-c?des=a3db76af0bcd91fa
LEF1 (Cell Signaling Technology 2230S, clone C12A5)
https://www.citeab.com/antibodies/123056-2230-lef1-c12a5-rabbit-mab?des=82bb7bda0df462a4
GSK3β (Bioworld BS6886)
https://www.citeab.com/antibodies/2208670-bs6886-gsk3-polyclonal-antibody?des=521c8eba99dd398d
Phospho-GSK-3β (Cell Signaling Technology 5558S, clone D85E12)
https://www.citeab.com/antibodies/125495-5558-phospho-gsk-3-ser9-d85e12-xp-rabbit-mab?des=347f35a0d20eb7ed
β-catenin (Cell Signaling Technology 8480S, clone D10A8)
https://www.citeab.com/antibodies/125282-8480-catenin-d10a8-xp-rabbit-mab?des=c2a0da8083843490
p-β-catenin(S33/37/T41) (Cell Signaling Technology 9561S)
https://www.citeab.com/antibodies/126241-9561-phospho-catenin-ser33-37-thr41-antibody?des=2e54a02d7dc56fe8
GAPDH (Proteintech HRP-60004, clone 1E6D9) https://www.citeab.com/antibodies/982052-hrp-60004-gapdh-antibody?des=8231d0723cc00938
GAPAH (Cell Signaling Technology 8884S, clone D16H11)
https://www.citeab.com/antibodies/125652-8884-gapdh-d16h11-xp-rabbit-mab-hrp-conjugate?des=d195db4441265b95
TGFBI (Cell Signaling Technology 5601S, clone D31B8)
https://www.citeab.com/antibodies/125526-5601-ig-h3-d31b8-xp-rabbit-mab?des=5d59e5d8cf62ac5d
Laminin (Abcam ab78286, clone P3H9-2)
https://www.citeab.com/antibodies/741580-ab78286-anti-laminin-5-antibody-p3h9-2?des=cc12924e1fdb60c2
PCNA (Cell Signaling Technology 2586S, clone PC10)
https://www.citeab.com/antibodies/123622-2586-pcna-pc10-mouse-mab?des=7db095096d839dfb

Eukaryotic cell lines

Policy information about cell lines

Cell line source(s) — The human iPSC line was purchased from Nuwacell Biotechnologies (hiPSC, RC01001-B, 593 Female, Nuwacell

Cell line source(s)	Biotechnologies Co., Ltd).<br>The HEK 293T cell line (3101HUMGNHu17) and HaCaT cell line (1101HUM-PUMC000373) was obtained from the Cell Resource Center, Peking Union Medical College.
Authentication	Authentication was conducted by each manufacturer and each cell line we used was morphologically confirmed.
Mycoplasma contamination	All cell lines tested negative for mycoplasma.
Commonly misidentified lines (See ICLAC register)	None.

Animals and other organisms

Policy information about studies involving animals; ARRIVE guidelines recommended for reporting animal research

Laboratory animals	C57BL/6 mice (male, 6-8 weeks old) were purchased from Beijing Vital River Laboratory Animal Technology Co., Ltd. (Beijing, China). Mice were housed in an animal facility under constant environmental conditions (room temperature, 20-25°C; relative humidity, 40-60% and a 12-h light-dark cycle).
Wild animals	No wild animals used.
Field-collected samples	This study did not involve animals collected from the field.
Ethics oversight	All animal experiments were performed following standards with the approval of the Institutional Animal Care and Use Committee at the National Center for Protein Sciences (Beijing).

Note that full information on the approval of the study protocol must also be provided in the manuscript.

Human research participants

Policy information about studies involving human research participants

Population characteristics	Discarded human foreskin tissues were obtained (anonymopusly) from healthy donors (aged between 18 and 65 years) undergoing circumcision as normal control tissues. The foreskin tissues of Secondary syphilis were obtained (aged between 18 and 65 years) from skin biopsy. The detailed information was provided in Supplementary Table 1.
Recruitment	Skin samples were recruited through the Peking Union Medical College Hospital, China. Signed informed consents were obtained from all donors prior to the study. There was no self-isolation bias or any other bias for recruiting donors to the study.
Ethics oversight	Written informed consent was obtained from all the participants. This study was approved by the institutional review board of the Peking Union Medical College Hospital (reference number ZS-2556).

Note that full information on the approval of the study protocol must also be provided in the manuscript.
